# Bias and inconsistency in the estimation of tumour mutation burden

**DOI:** 10.1186/s12885-022-09897-3

**Published:** 2022-08-02

**Authors:** Mohammad A. Makrooni, Brian O’Sullivan, Cathal Seoighe

**Affiliations:** School of Mathematical &amp; Statistical Sciences, National University of Ireland, Galway, Ireland

**Keywords:** Tumour mutation burden, Variant allele frequency, Tumour heterogeneity

## Abstract

**Background:**

Tumour mutation burden (TMB), defined as the number of somatic mutations per megabase within the sequenced region in the tumour sample, has been used as a biomarker for predicting response to immune therapy. Several studies have been conducted to assess the utility of TMB for various cancer types; however, methods to measure TMB have not been adequately evaluated. In this study, we identified two sources of bias in current methods to calculate TMB.

**Methods:**

We used simulated data to quantify the two sources of bias and their effect on TMB calculation, we down-sampled sequencing reads from exome sequencing datasets from TCGA to evaluate the consistency in TMB estimation across different sequencing depths. We analyzed data from ten cancer cohorts to investigate the relationship between inferred TMB and sequencing depth.

**Results:**

We found that TMB, estimated by counting the number of somatic mutations above a threshold frequency (typically 0.05), is not robust to sequencing depth. Furthermore, we show that, because only mutations with an observed frequency greater than the threshold are considered, the observed mutant allele frequency provides a biased estimate of the true frequency. This can result in substantial over-estimation of the TMB, when the cancer sample includes a large number of somatic mutations at low frequencies, and exacerbates the lack of robustness of TMB to variation in sequencing depth and tumour purity.

**Conclusion:**

Our results demonstrate that care needs to be taken in the estimation of TMB to ensure that results are unbiased and consistent across studies and we suggest that accurate and robust estimation of TMB could be achieved using statistical models that estimate the full mutant allele frequency spectrum.

**Supplementary Information:**

The online version contains supplementary material available at (10.1186/s12885-022-09897-3).

## Background

Immunotherapy is an evolving and promising cancer treatment that works by provoking the patient’s own immune response to target cancer cells [1]. Several studies have examined the effectiveness of Immunotherapy drugs in cancer treatment [2–4]. Although many patients show a strong and durable response to immune checkpoint inhibitors (ICIs), some patients do not respond well to treatment [5, 6]. Therefore, there is a need for effective biomarkers to distinguish between patients who are more or less likely to benefit from this treatment. Several biomarkers have been proposed that correlate well with the response of immunotherapy in multiple cancer types, including tumour mutation burden (TMB), neoantigen burden, DNA mismatch repair deficiency, and high microsatellite instability [7–11].

TMB was initially introduced as a biomarker for ICIs in melanoma. Recently the FDA has approved pembrolizumab in all cancers with TMB greater than 10 mutations per megabase (MB) as assessed by the targeted FoundationOne CDx assay [12]. Several studies have argued that patients with high tumor mutation burden (TMB-H) are more likely to respond to checkpoint inhibitors because a higher number of mutations in a tumour correlates with an increase in the number of neoantigens that can be recognized by T cells [13–15]. However, some recent studies have shown that TMB-H fails to predict immune checkpoint blockade response in breast cancer, prostate cancer and glioma [16, 17]. Despite the low efficacy of TMB in these cancer types, significant correlations have been reported between TMB-H and response to ICIs in several other cancer types such as melanoma, lung, and bladder cancers where CD8 T-cell levels positively correlated with neoantigen load ([16, 18]). TMB-H has been reported to be the most robust, effective and clinically verifiable biomarker in these cancer types [19].

Tumour mutation burden is calculated by counting the number of somatic mutations above a threshold frequency in data derived from whole genome sequencing, whole exome sequencing (WES) or panel sequencing and dividing by the size of the target region [20]. Although WES is frequently used to measure TMB in a research setting, it can be impractical for clinical use due to its higher cost, and the low average coverage which could result in missing rare somatic mutations. To overcome these issues some studies suggest using panel sequencing [21, 22]. The US FDA approved two cancer-related gene panels, FoundationOne CDx (F1CDx) and MSK-IMPACT [23, 24]. Also [19, 25] proposed pan-cancer TMB panels that showed higher correlation with WES than other panels. Several studies have suggested using various thresholds, filtering strategies and models to improve the robustness of TMB measurement for targeted-panel sequencing and to correct panel design biases in order to avoid overestimating TMB [26–29]. To validate the panel-based sequencing approach, the TMB derived from the target-panel is compared against TMB measured from WES [30], which is considered the standard TMB measurement [31].

Although TMB is an effective biomarker in several cancer types and the efficacy of panel based sequencing in TMB estimation has been shown in several studies as an alternative to WES sequencing for clinical use, there is still no standard method to determine the genes to include in the panel, the type of mutation or the cut-off to distinguish between high and low TMB values [32, 33]. Apart from the lack of a standardization method which is essential in order to be able to compare the different TMB values estimated from different gene panels, there are some factors that could influence TMB estimation regardless of the NGS platform used. These factors could be categorized into two groups, patient and sample-specific factors, such as the site of biopsy, sample type, sample purity and technical factors, such as sequencing depth and bioinformatics pipeline [34–39].

The first step in a TMB estimation pipeline is to detect somatic mutations. Many mutation calling tools have been introduced to call somatic single nucleotide variants and the performance of these tools has been evaluated extensively [40–42]. In particular, [43] showed that Mutect2, developed by the Broad Institute, EBCall [44], Virmid [45] and Strelka [46] are the most reliable tools, with similar performance. Several studies have also evaluated the effect of sequencing depth in detecting somatic mutations by different mutation callers. In these studies, in particular [47], it has been shown that a sequencing depth ≥200*X* is sufficient for calling 95% of mutations with mutation frequency (≥20*%*); for mutations at lower frequencies, it has been recommended to increase the sequencing depth or improve the experimental method. Therefore in the absence of sufficient coverage depth, detecting somatic variant with low frequency is still a major challenge. Insufficient sequencing depth could also impact on TMB estimation by reducing the accuracy of mutation frequency estimates as the mutation frequency is required in order to determine the number of mutations exceeding the threshold frequency.

In this study, we investigate statistical biases affecting TMB estimation. We explore the impact of these biases on TMB estimates using simulations with parameters informed by real cancer sequencing studies. We also investigate the relationship between inferred TMB and sequencing depth, both by down-sampling sequencing reads from exome sequencing datasets from TCGA and by assessing the relationships between inferred TMB and sequencing depth across TCGA cohorts. The relationship between sequencing depth and inferred TMB is likely to reflect both the power to detect somatic mutations [47] as well as bias in the TMB estimates, which is also a function of sequencing depth. We suggest that a statistical modelling approach that estimates the parameters of the entire mutation frequency spectrum, rather than counting mutations above a fixed threshold, is likely to provide a more robust means of estimating TMB.

## Methods

### Simulations

**Fig. 1:**
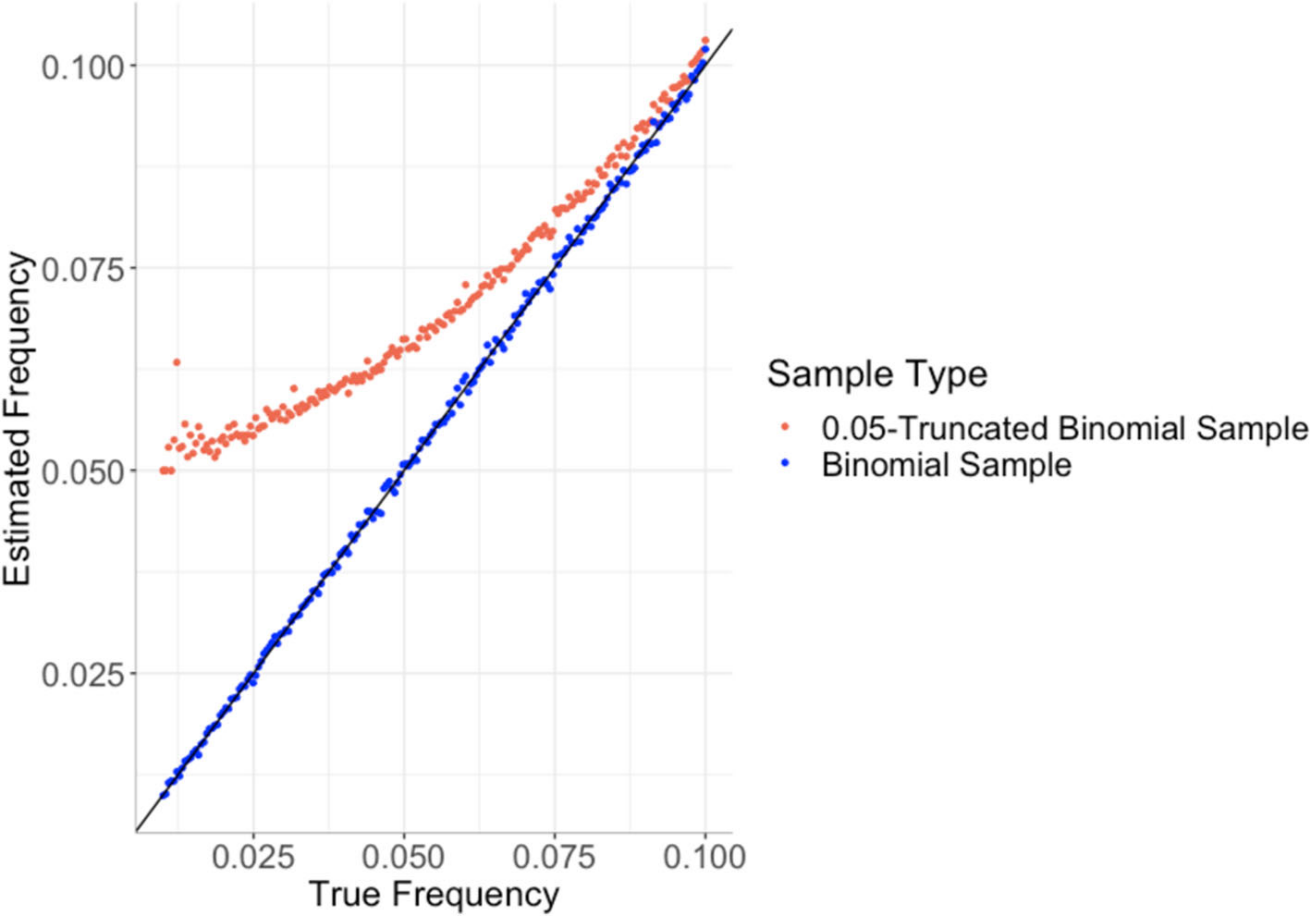
The estimated frequencies (the proportion of successes) versus the true frequencies (the success probability) for both binomial (blue) and 0.05-truncated binomial (red) random variables

We used simulations to investigate the extent of the bias in the estimate of mutant allele frequencies resulting from neglecting mutations for which the empirical frequency was below a threshold, *τ*. The simulations consisted of 200 loci, each covered by 100 reads. True values of the mutant allele frequency were considered, over the range shown in Fig. 1. For each true frequency, *f*, we obtained 1,000 random samples from a binomial random variable, with parameter, *f* and size 100, using R. Two estimates were then returned for the mutant allele frequency, one derived from all of the samples and a second, following truncation (i.e. using only the samples in which the proportion of mutant alleles was at least *τ*).

### Theoretical derivation of the relative error in the TMB estimates

**Fig. 2:**
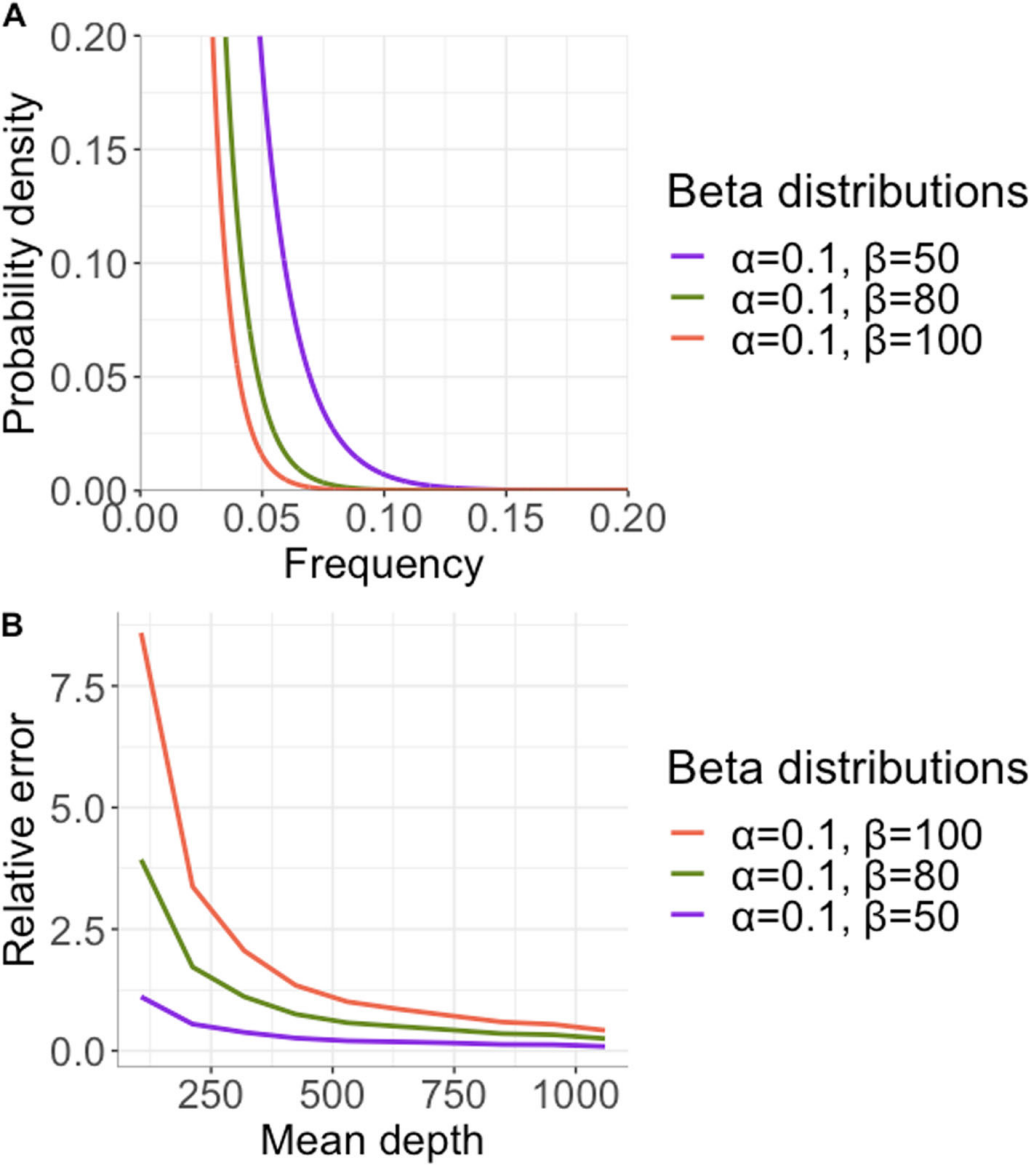
**A**. Three beta distributions, with *α* and *β* parameters as shown in the legend, representing alternative mutant allele frequency spectra for subclonal mutations. **B**. Relative error in the estimated TMB contribution from subclonal mutations derived from the three distributions in A

Here we provide a theoretical derivation of the extent of the bias in TMB corresponding to subclonal mutations with beta-distributed mutant allele frequency spectrum, with parameters *α* and *β*, as illustrated in Fig. 2. The true number of somatic mutations with frequency above *τ* is

1 $$ T=\left(1-{F}_B\left(\tau; \alpha, \beta \right)\right)\times \mathrm{S}. $$

where *F*_*B*_ is the cumulative distribution function of a beta random variable and *S* is the total number of somatic mutations.

Let *d*_*i*_ be the sequencing depth at a site *i* at which a somatic mutation has occurred. The number of reads carrying the mutant allele at site *i* is a beta-binomial random variable with size *d*_*i*_ and parameters *α* and *β* and the expected number of somatic mutations with frequency ≥*τ* is:

2 $$ E=\sum \limits_{i=1}^S\left(1-{F}_{BB}\left({d}_i\tau; {d}_i,\alpha, \beta \right)\right). $$

where *F*_*BB*_ is the cumulative distribution function of the beta-binomial random variable. The relative error (depicted in Fig. 2) is (*E*−*T*)/*T*.

### Down-sampling TCGA data and TMB calculation

Whole exome sequencing data (in BAM format) was downloaded for four lung adenocarcenoma (LUAD) TCGA samples from cBioPortal ([48]); TCGA-55-8205, TCGA-78-7159, TCGA-78-7161 and TCGA-78-7162). We used samtools to downsample each BAM file progressively to 50%. We used Mutect2, GATK4 with the default options to infer somatic mutations and their frequencies. To estimate TMB we determined the number of PASS somatic mutations with estimated frequencies above 0.05.

### Correlation between sequencing depth and TMB

We analyzed paired tumour-normal whole-exome sequencing data from 4,850 TCGA samples from ten primary tumour types (bladder urothelial carcinoma; *N*=411 sample pairs, breast invasive carcinoma; *N*=1,043, colon adenocarcinoma; *N*=432, kidney renal clear cell carcinoma; *N*=338, brain lower grade glioma; *N*=511, lung adenocarcinoma; *N*=569, lung squamous cell carcinoma; *N*=496, ovarian serous cystadenocarcinoma; *N*=440, prostate adenocarcinoma; *N*=496 and skin cutaneous melanoma; *N*=104). The number of mapped reads from the tumour BAM for each donor was used as a proxy for sequencing depth. In order to observe how tumour heterogeneity affects the relationship between sequencing depth and TMB, we used TCGA samples from all cancer groups that are present within PCAWG data, for which the proportion of clonal mutations in each sample is known. These files were downloaded from ICGC ([49]) and access was granted through DACO-5661 and dbGAP Project 21959. In each cancer cohort we calculated the proportion of samples with high clonal fraction (i.e. the proportion of the samples with clonal fraction greater than 50%).

## Results

### The proportion of reads corresponding to a somatic mutation is a biased estimator of mutation frequency

To calculate the TMB a tumour sample is obtained and the genome (or a targeted subset of the genome, such as the exome or a gene panel) is sequenced, usually to a relatively high-depth. In the first instance, we make the simplifying assumptions that all somatic mutations present in the sequence reads can be detected with perfect efficiency (i.e. we neglect the effects of sequencing and mapping errors and any other artefacts) and that a sample consisting only of cancer cells has been sequenced to depth of *N* reads (constant across sites). We wish to obtain an estimate of the TMB, defined as the number of somatic mutations per megabase whose true frequencies are above a threshold, *τ*. This is estimated by counting the mutations with frequency above *τ* and dividing by the size of the target region. This approach requires the mutant allele frequency to be estimated (to determine whether it exceeds *τ*). The proportion of reads containing the mutant allele is used as an estimate of the true mutation frequency, *f* ([50]); however, in this case the proportion of reads containing the mutant allele is a biased estimator of *f*. This bias results from the fact that the proportion is calculated only for sites at which at least one mutant allele is observed, whereas the true set of sites at which a somatic mutation has occurred is unknown (and may be much larger [47]). The number of mutant alleles observed among the sequence reads at a site is, therefore, a sample from a zero-truncated binomial random variable [51]. The expected value of the proportion of successes from a zero-truncated binomial random variable is $\frac {f}{1-(1-f)^{N}}$, which exceeds *f* (Fig. S1).

Fisher derived a maximum likelihood procedure to estimate the parameter *f* in a singly truncated binomial distribution [51]. The extent of the bias resulting from zero-truncation depends on the true (but unknown) frequency spectrum of the somatic mutations in the sample and is largest when there are many low-frequency somatic mutations. In practice, the bias is much larger than shown in Fig. S1, because in the calculation of TMB, typically only sites at which the observed proportion of reads containing the mutant allele is greater than the threshold *τ* (often set at 0.05) are considered. The number of reads with the alternative allele is therefore a sample from an *τN*-truncated binomial random variable and the upward bias in the estimate of the true mutation frequency has the potential to be substantial (Fig. 1).

### Bias in TMB resulting from uncertainty in frequency estimates

**Fig. 3:**
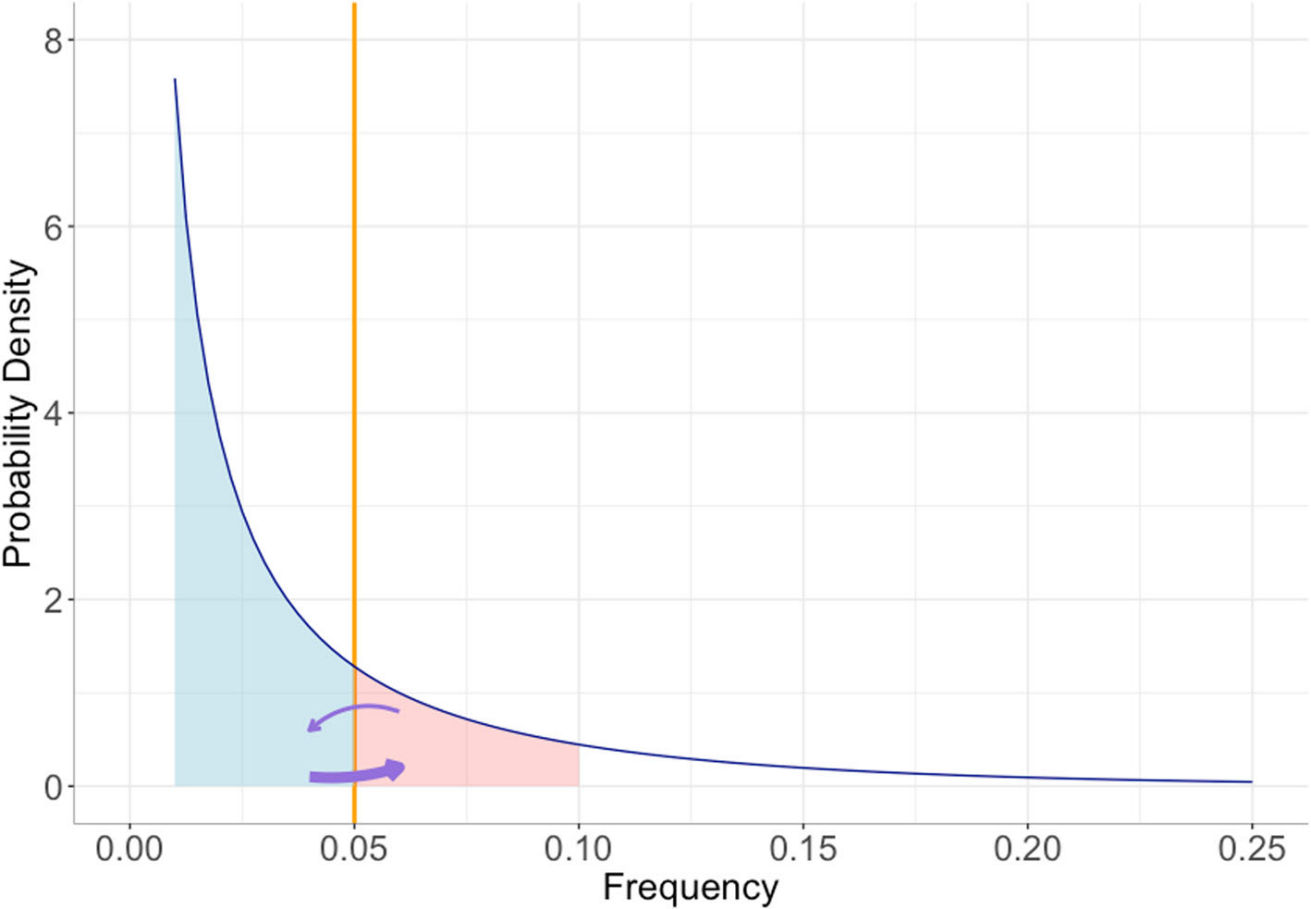
Illustration of how uncertainty in mutation frequency estimates can lead to over-estimation of the number of mutations above the frequency threshold, even if the estimated frequencies are unbiased. The red and blue shaded areas correspond to mutations for which sampling error could cause them to cross the frequency threshold (i.e. the estimated frequencies of mutations with true frequencies in the red shaded area may be below the threshold due to sampling error, while the estimated frequencies of mutations in the blue shared area may be above the threshold). Because the blue shaded area is much larger than the red area, the number of mutations that pass the threshold from left to right is likely to be much larger than the number of mutations that pass the threshold in the other direction, leading to over-estimation of the number of mutations above the threshold

Even if the complete set of sites at which a somatic mutation has occurred were known, so that the mutation frequencies were not affected by truncation bias, the number of mutations above the frequency threshold is likely to be a biased estimate of the TMB. This is because in addition to bias, the mutation frequency estimates include uncertainty. If we count the number of mutations with empirical proportions greater than *τ* and if the mutation frequency spectrum has a strongly negative slope at *τ* then the number of mutations with true frequency below *τ* but empirical proportion above *τ* (i.e. moving from left to right across the yellow line in Fig. 3, Fig. S2) may be much larger than the number passing the threshold in the other direction, resulting in over-estimation of TMB. As an example, Fig. S3 shows the points that cross the threshold to the left and right for a simulation using *α*=0.1 and *β*=100.

Models of exponential growth and largely neutral tumour evolution predict a large number of low-frequency variants [52] and simulations suggest that the two sources of bias introduced in this and the previous section can result in substantial bias in TMB estimated from cancer samples, with the impact on TMB depending on the shape of the mutant allele frequency spectrum (Fig. 2 and Fig. S3).

### Relationship between coverage and TMB in 10 different cancer cohorts

**Fig. 4:**
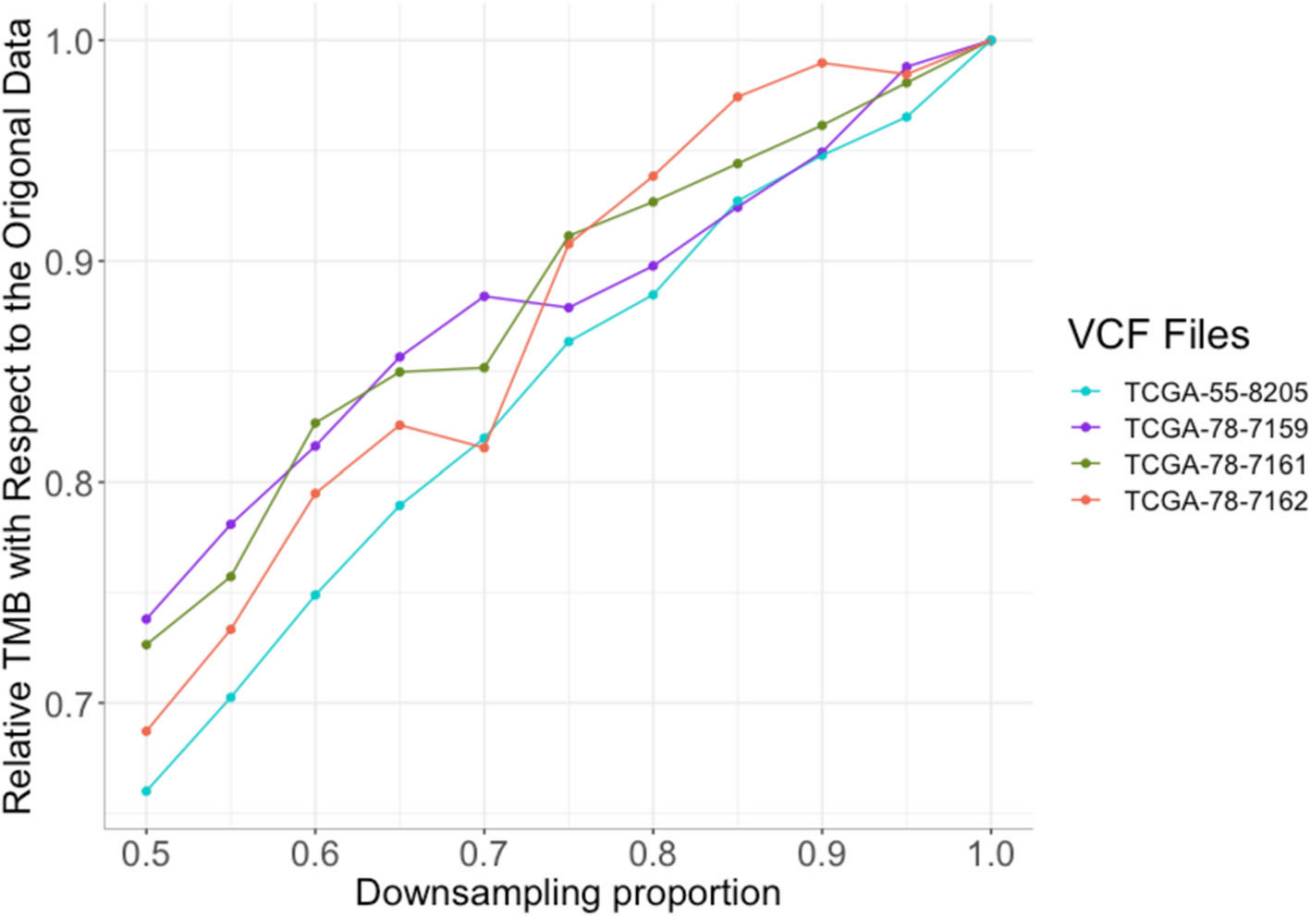
Each TCGA data was downsampled to 50 percent. The plot shows the TMB in each downsampled data with respect to its corresponding original full data

The above demonstration assumes perfect power to identify the somatic mutations on the sequenced reads. In reality, many of the somatic mutations present on the sequenced reads may not be identified by somatic mutation calling pipelines. The power to detect a somatic mutation at a site will depend on the sequencing depth and depth also influences the extent of the bias resulting from *τN*-truncation. The combination of these effects is likely to result in instability in the TMB estimate as the sampling depth is varied, potentially resulting from inconsistent results obtained by different experimental protocols. To illustrate this potential instability we down-sampled the sequencing reads from real data (from TCGA) to 50% of their sequencing depths and implemented a pipeline to estimate TMB (with a frequency threshold of 0.05). The TMB estimated in this way was sensitive to sequencing depth following down sampling and showed no evidence of having reached a plateaux by the time the full sample depth was included (Fig. 4). To investigate the relationship between sequencing depth and TMB across real exome sequencing data we analyzed 4,850 TCGA samples from ten cancer types. In six of the cancer types there was a statistically significant positive correlation between sequencing depth and TMB, although the depth appears to explain only a small proportion of the variation in TMB (Fig. S5).

## Discussion

The number of somatic mutations observed in tumours has been studied extensively in recent years to understand its efficacy as a predictive biomarker in different cancer types as well as the factors that contribute to its variation between individuals and across cancer types [16, 17, 34–36]. To estimate TMB mutation callers are used to identify somatic variants and to estimate their frequencies. The number of somatic mutations above a specified threshold frequency per megabase of the genomic region targeted in the experiment is then reported as the TMB. The performance of the mutation callers depends on sequencing depth and mutation frequency and many mutations with low frequencies (≤10*%*) may be missed by mutation callers at moderate sequencing depths [47], potentially leading to underestimation of TMB. Unbiased estimation of TMB also requires unbiased frequency estimation. In this study, we report two sources of bias in TMB estimation that can lead to incorrect TMB estimates and inconsistency across studies.

The first source of bias results from misestimation of somatic mutation frequencies. The number of mutant alleles obtained when a genomic site is sequenced at some depth is a binomial random variable. However, if only sites at which a mutation is observed are considered, then this random variable is zero-truncated. In the early part of the last century Fisher showed that the proportion of successes obtained from a zero-truncated random variable is a biased estimate of the success probability [51]. This bias becomes more severe if the distribution is truncated at a frequency above zero, as is the case in the calculation of TMB. The second source of bias is due to the uncertainty in the estimated frequency. Even if the frequency estimate is unbiased, the number of somatic mutations with estimated frequency above a given threshold may be a biased estimate of the number of mutations with true frequencies above the threshold. This is because the number of somatic mutations with true frequencies to the left or right of the threshold may be very different, as illustrated in Fig. 3. The extent to which these two sources of bias affect TMB estimates depends on the shape of the variant allele frequency spectrum and can be substantial if there is a high proportion of low-frequency subclonal variants, with a steep slope in the variant allele frequency spectrum around the threshold (Fig. 2).

In the results based on simulated data the locations of all somatic mutations were known. In real data the somatic mutations must be inferred from mapped sequencing reads and they are not recovered with perfect efficiency, so that estimated TMB will be a function of both the power to detect somatic mutations as well as the biases, described above, that affect the number of mutations with observed frequency above the threshold. The down-sampling experiments we carried out were intended to assess the combined effects of these factors as a function of sequencing depth. We observed that the TMB is not consistent across different sequencing depths (Fig. 4). We also analyzed 10 TCGA cancer cohorts to assess the relationship between TMB and sequencing depth in real cancer samples. Our results showed that, in six out of the ten cancer types, TMB and sequencing depth are positively correlated (consistent with Fig. 4). The influence of tumour purity on mutation detection in mutation caller tools has been studied previously [53] and it is well known that the presence of normal cells in tumour samples can result in underestimation of tumour mutation allele frequencies [50], hence impacting on TMB estimation. A positive correlation has previously been reported between sample purity and TMB [36] and methods have been suggested to account for tumour purity in calculation of TMB, such as increasing sequencing depth and dividing variant allele frequency (VAF) by purity and increasing the threshold [54]. Given that the majority of samples we studied have high purity (above 50%), our results suggest that sequencing depth can have an impact on the TMB even in samples with high purity (Fig. S6).

Although many of the cancer types showed evidence of correlation between sequencing depth and TMB, this was not the case for bladder urothelial carcinoma (BLCA), lung adenocarcinoma (LUAD), prostate adenocarcinoma (PRAD), skin cutaneous melanoma (SKCM). Using PCAWG data in which clonal and subclonal mutations have been distinguished, we found that the lack of correlation between TMB and coverage in some cancer types is likely due to the high clonal proportions in samples within these cancer types (Fig. S7). Because they occur at high frequencies (far in excess of the frequency thresholds used to define TMB), clonal mutations are easier to detect and contribute unambiguously to the count of mutations exceeding the frequency threshold. Unsurprisingly, more heterogenous tumours (with high proportions of subclonal mutations) are more likely to be influenced by changes in sequencing depth. This is consistent with the observation of higher TMB in metastatic cancers, which has been suggested to result from bottlenecks in cell populations leading to increased proportions of clonal variants [55, 56]. Therefore, tumour heterogeneity may impact TMB estimates and may explain some of the variability in estimated TMB values across studies. Interestingly, tumour heterogeneity has also been suggested as a companion to TMB to achieve better performance in ICI response prediction ([57]).

Our study demonstrates that there can be substantial biases in TMB estimates when the mutational burden includes a large contribution from subclonal mutations. These biases result from lack of power to detect low-frequency variants as well as bias and uncertainty in estimated mutation frequencies. There are at least two ways in which this issue can be addressed. At higher sequencing depths the power to detect low-frequency variants increases. Given any TMB threshold it is possible to determine the sequencing depth that would be required to achieve higher power to recover somatic mutations at or above that threshold. Although, the biases we describe here also decrease with increasing sequencing depth it is less easy to determine the relationship between sequencing depth and the bias in TMB resulting from these effects as they depend on the shape of the mutation frequency spectrum. An alternative approach, which may provide stable estimates of TMB even for lower sequencing depths, would be to use all of the data generated to estimate the shape of the variant allele frequency spectrum and, from this, to derive an estimate of the TMB. Although in-principle this is possible, it will require the development of sophisticated statistical models that can account appropriately for all technical factors that can influence the probability with which somatic mutations are recovered and their observed frequency in tumour sequencing data.

## Conclusion

We have examined two sources of bias that can affect current methodologies to estimate TMB. The impact of these biases depends on the mutant allele frequency spectrum and it can be substantial when the TMB includes a large contribution from subclonal mutations. These strength of these biases, as well as the power to detect subclonal mutations, vary with sequencing depth, resulting in the potential for inconsistency in TMB estimated using different sequencing depths. We show through an analysis of data from TCGA that there is a correlation between sequencing depth and estimated TMB, except in the case of tumours with large proportions of clonal variants. Overall, our findings caution that current methods to estimate TMB can be biased as well as inconsistent at different sequencing depths and we suggest that accurate and robust estimation of TMB could be achieved using statistical models to estimate parameters of the mutant allele frequency spectrum.

## Supplementary Information

**Additional file 1** Supplementary material. The additional file 1 contains figurers.

## Data Availability

The data that support the findings of this study are available from TCGA but restrictions apply to the availability of these data, which were used under license for the current study, and so are not publicly available. Data are however available from the authors upon reasonable request and with permission of TCGA.

## Abbreviations

TMB: Tumour Mutation Burden
ICI: Immune Checkpoint Inhibitors
FDA: Food and Drug Administration
CD8: cluster of differentiation 8
WES: Whole exome sequencing
NGS: Next generation sequencing
TCGA: The Cancer Genome Atlas
VAF: variant allele frequency
BAM: Binary Alignment Map
ICGC: International Cancer Genome Consortium
PCAWG: PanCancer Analysis of Whole Genomes
BLCA: Bladder Urothelial Carcinoma
BRCA: Breast invasive carcinoma
COAD: Colon adenocarcinoma
KIRC: Kidney renal clear cell carcinoma
LGG: Brain Lower Grade Glioma
LUAD: Lung adenocarcinoma
LUSC: Lung squamous cell carcinoma
OV: Ovarian serous cystadenocarcinoma
PRAD: Prostate adenocarcinoma
SKCM: Skin Cutaneous Melanoma
