# Supplementary material for: Bias and inconsistency in the estimation of tumour mutation burden
Source: BMC Cancer. 2022 Aug 2;22:840. doi: 10.1186/s12885-022-09897-3 (PMC9347149; doi:10.1186/s12885-022-09897-3)
Supplement: Supplementary file 1 — Additional file 1 Supplementary material. The additional file 1 contains figurers. [file 12885_2022_9897_MOESM1_ESM.pdf]

supplementary materials

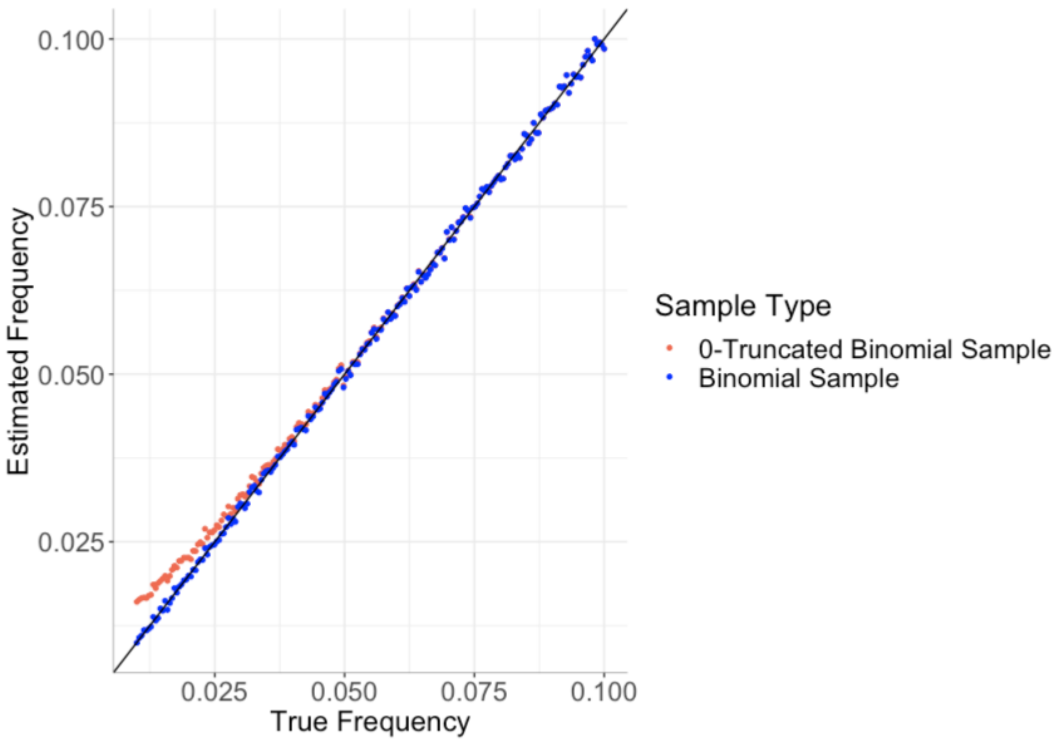

Fig. S1: Comparing the estimated frequencies, estimated by the proportion of alternative reads (or success) versus the true frequencies for both binomial (represented by the blue dots) and 0-truncated binomial samples (represented by the red dots). The plot suggests that the proportion of alternative reads is a biased estimator for 0-truncated binomial samples.

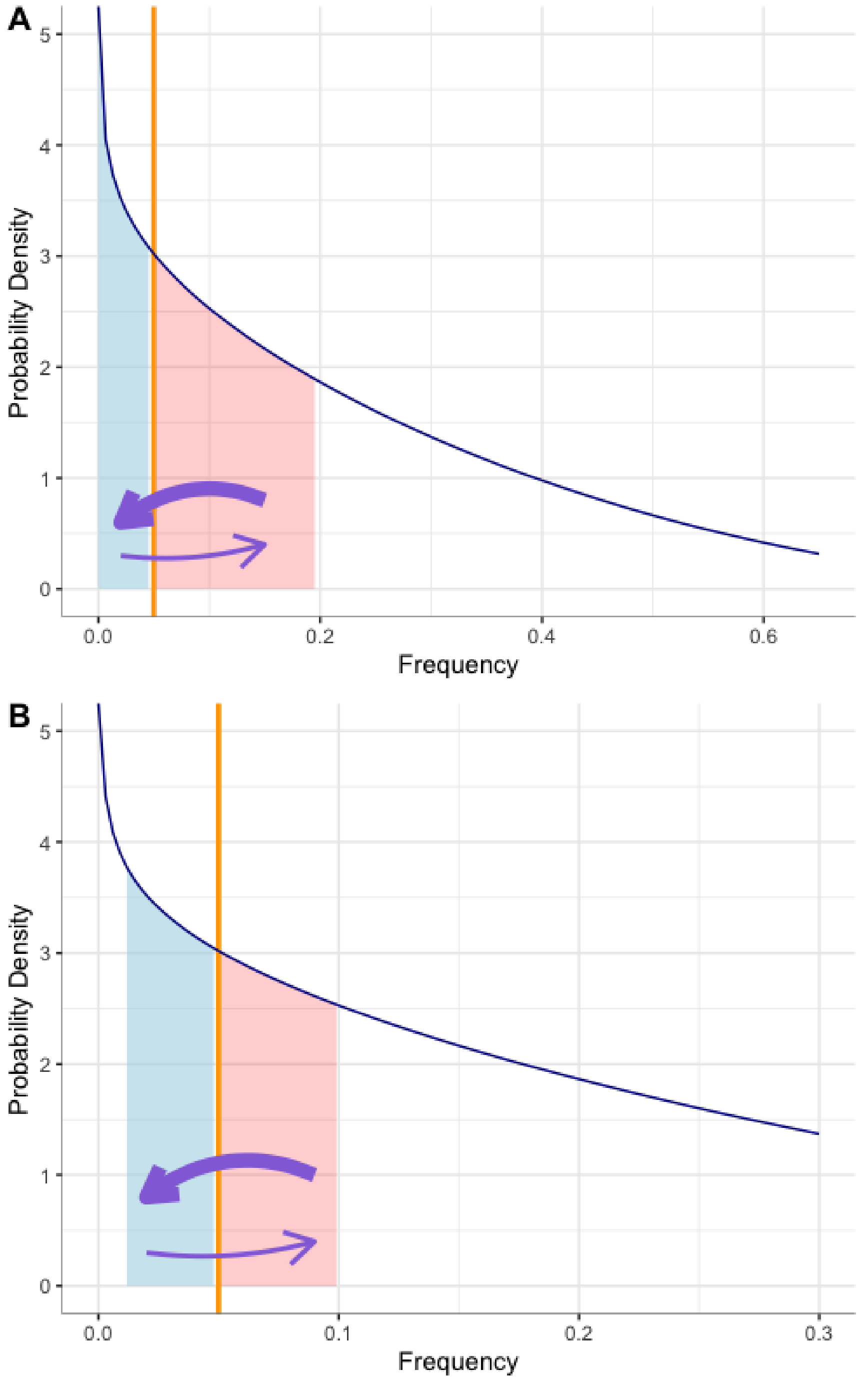

Fig. S2: Cases in which the counting method may underestimate TMB. The shaded red area represents the true frequencies (of somatic mutations in a tumor sample) that their corresponding estimated frequency might be less than 0.05. The blue area represents those true frequencies that their estimated frequency might cross the point of threshold. As the blue area is less than the red area, the plot suggests that the TMB estimated by counting the number of mutations with frequency above 0.05 may underestimates true TMB. However the underestimation error in B is smaller than A, as in B the tumor sample has been sequenced at higher depth which result in a smaller interval around the threshold that is not truncated on the left.

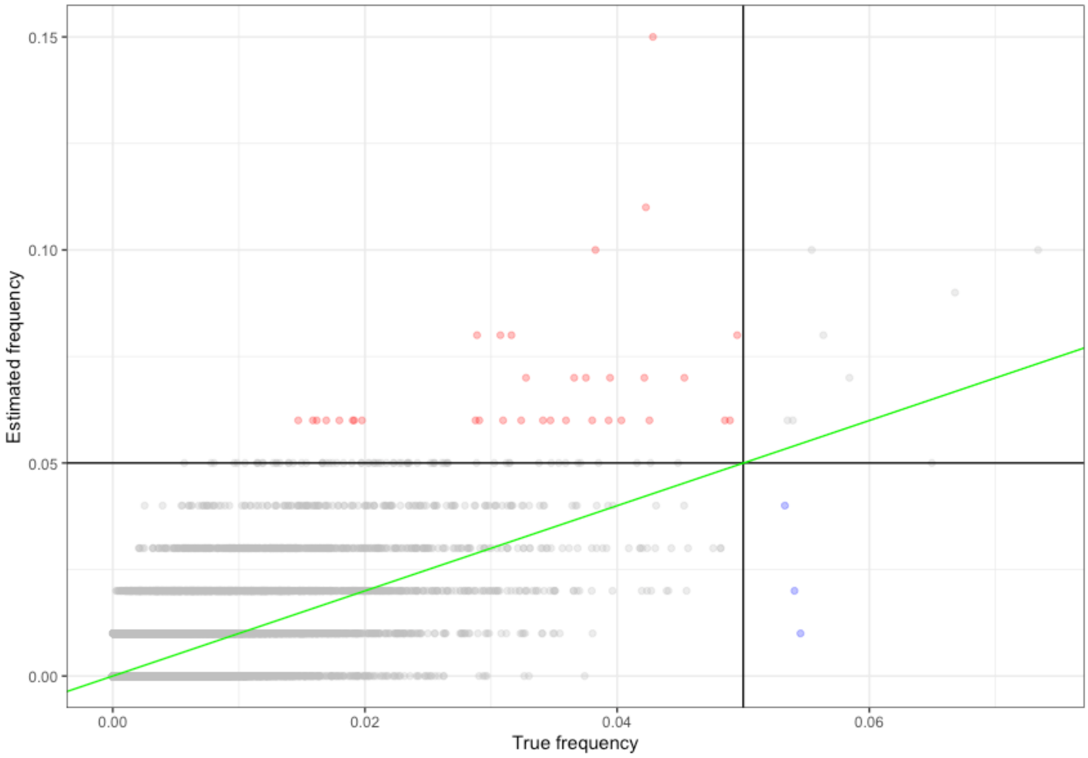

Fig. S3: The simulation shows the results of sampling random numbers from a beta distribution (with $\alpha = 0.1$ and $\beta = 100$, corresponding to a high density of low-frequency variants). The red points have true frequencies less than 0.05 and estimated frequencies greater than 0.05 (corresponding to points that moved from left to right in Figure. 3, i.e. into the red area depicted in the figure), while the blue points have moved across the threshold in the opposite direction (their true frequencies exceed the threshold, but their estimated frequencies are below the threshold; these points have moved into the region of the x-axis of Figure. 3 corresponding to the blue shading).

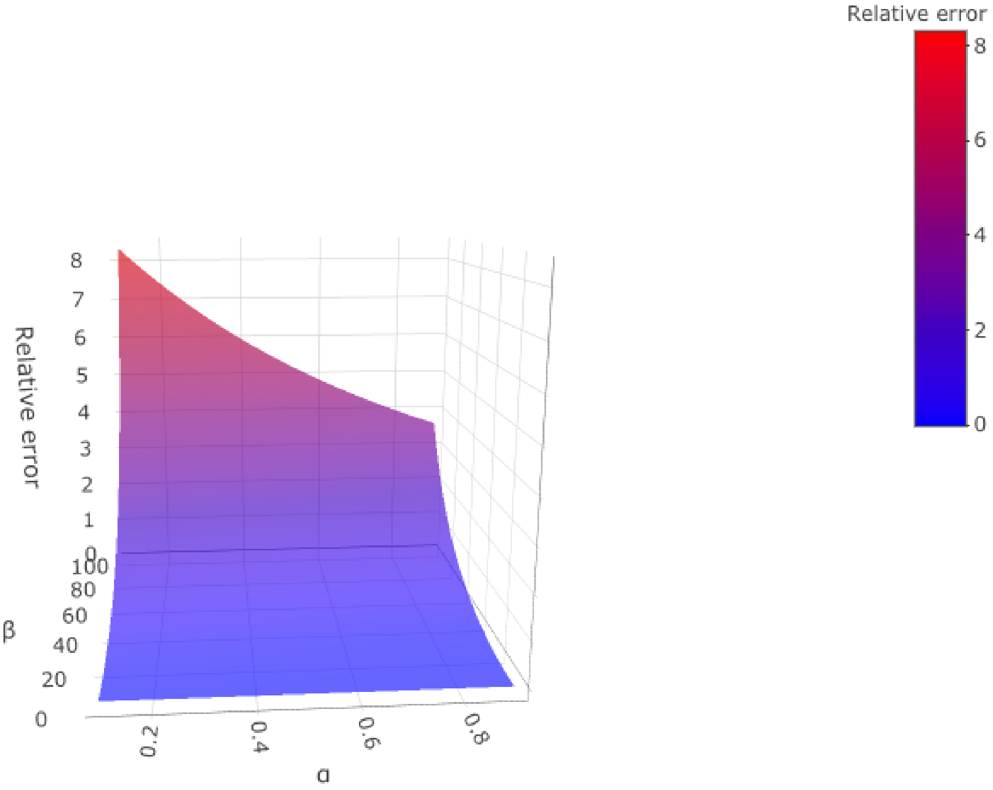

Fig. S4: The surface represents the relative error, calculated from our simulated data, over different parameter values. Positive relative errors correspond to overestimation. As we see, for beta distributions with parameter values within these ranges the amount of underestimation error is small, but in cases in which the beta density function is sharp the counting method may result in an overestimation error 8 times greater than the true TMB.

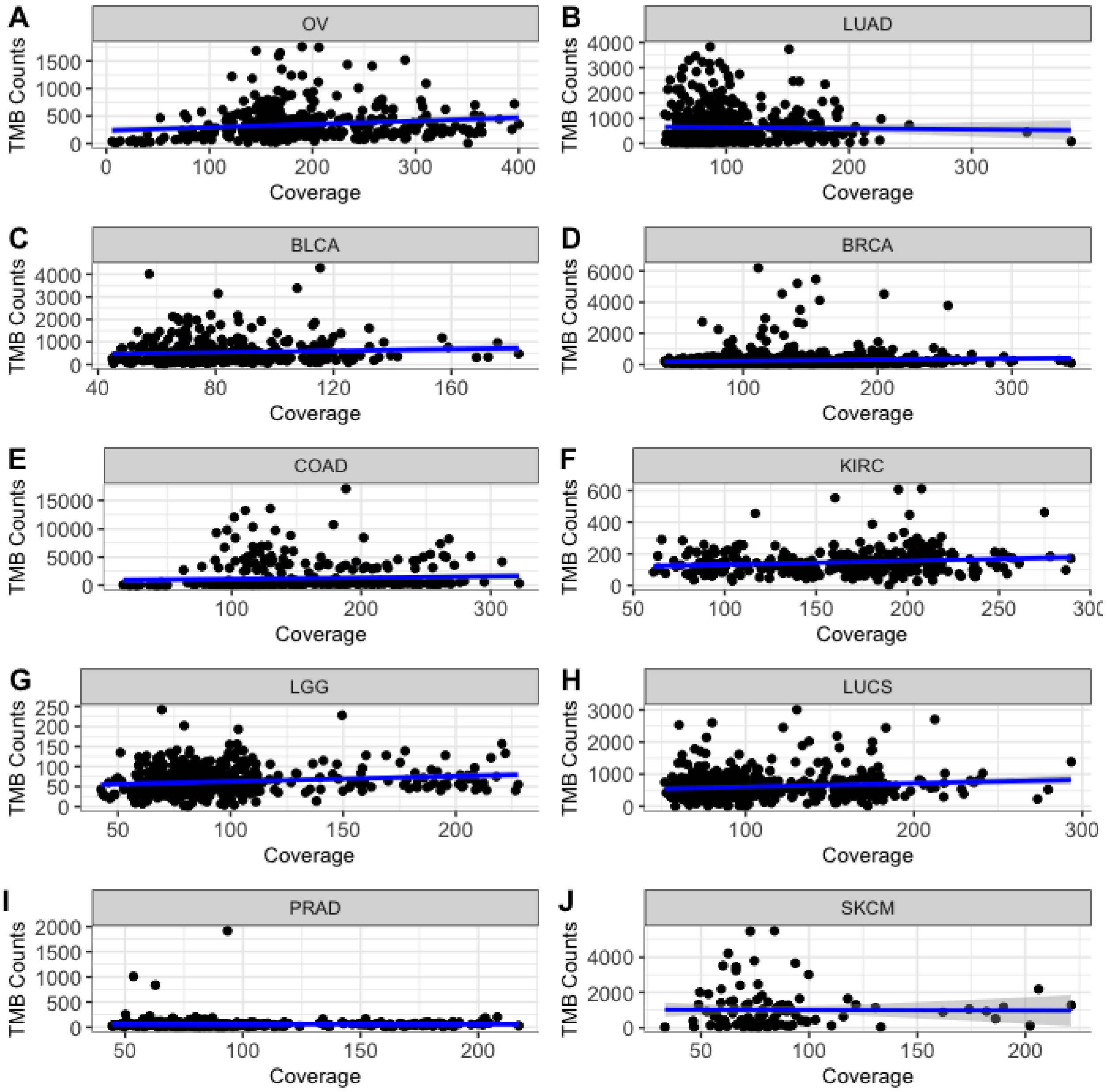

Fig. S5: (A-I), Spearman correlation analysis to test for relationship between TMB and coverage. The results show a positive relationship between Coverage and TMB for 6 out of the 10 cancer types. OV (Spearman ρ =0.15, p-value=0.00), LUAD (Spearman ρ =0.04, p-value=0.32), BLCA (Spearman ρ =0.08, p-value=0.07), BRCA (Spearman ρ =0.13, p-value=0.00), COAD (Spearman ρ =0.27, p-value=0.00), KIRC (Spearman ρ =0.16, p-value=0.00), LGG (Spearman ρ =0.15, p-value=0.00), LUSC (Spearman ρ =0.13, p-value=0.00), PRAD (Spearman ρ =0.02, p-value=0.57), SKCM (Spearman ρ =0.07, p-value=0.47)

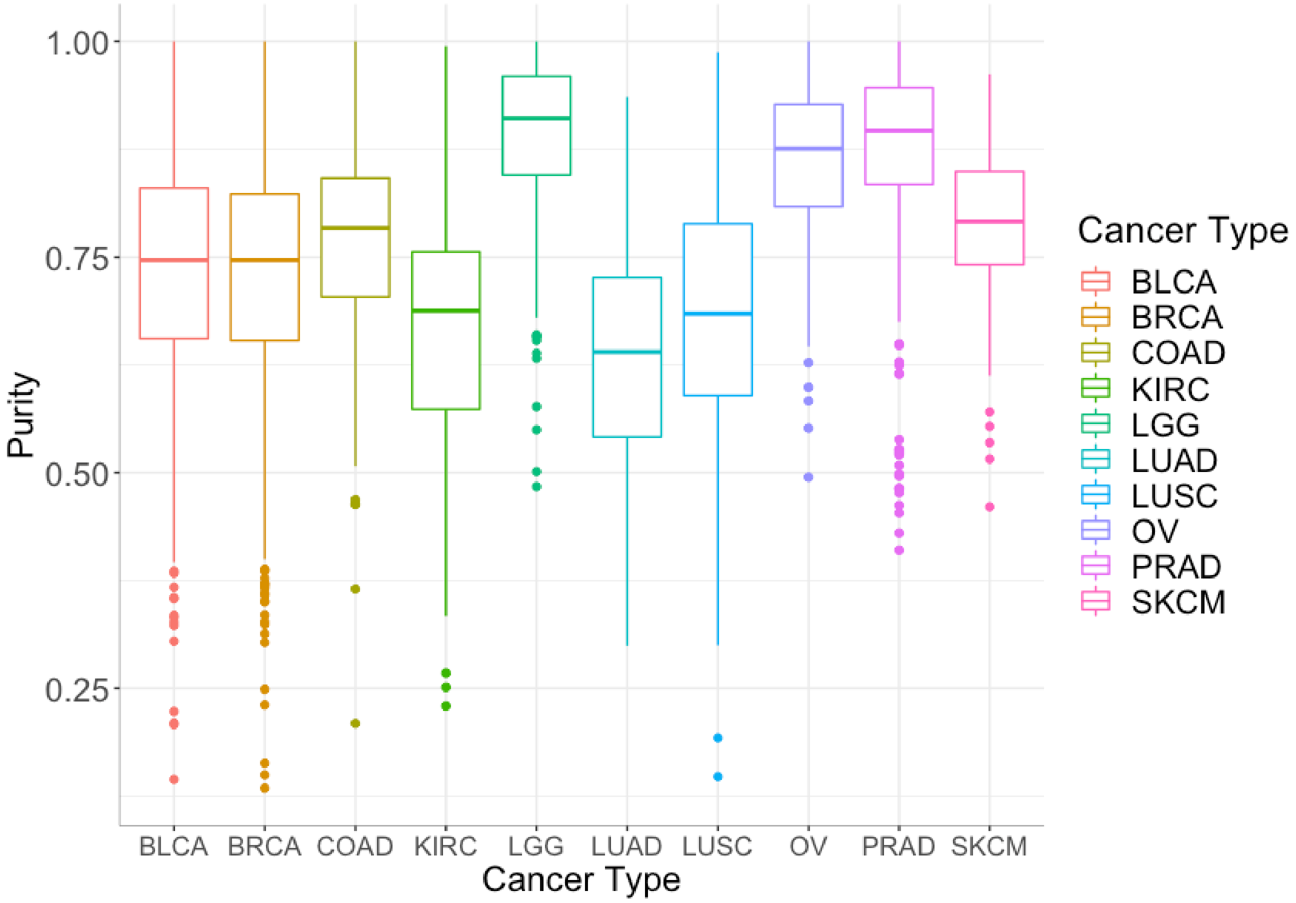

Fig. S6: Boxplot of sample purities across 10 cancer cohorts used in this study.

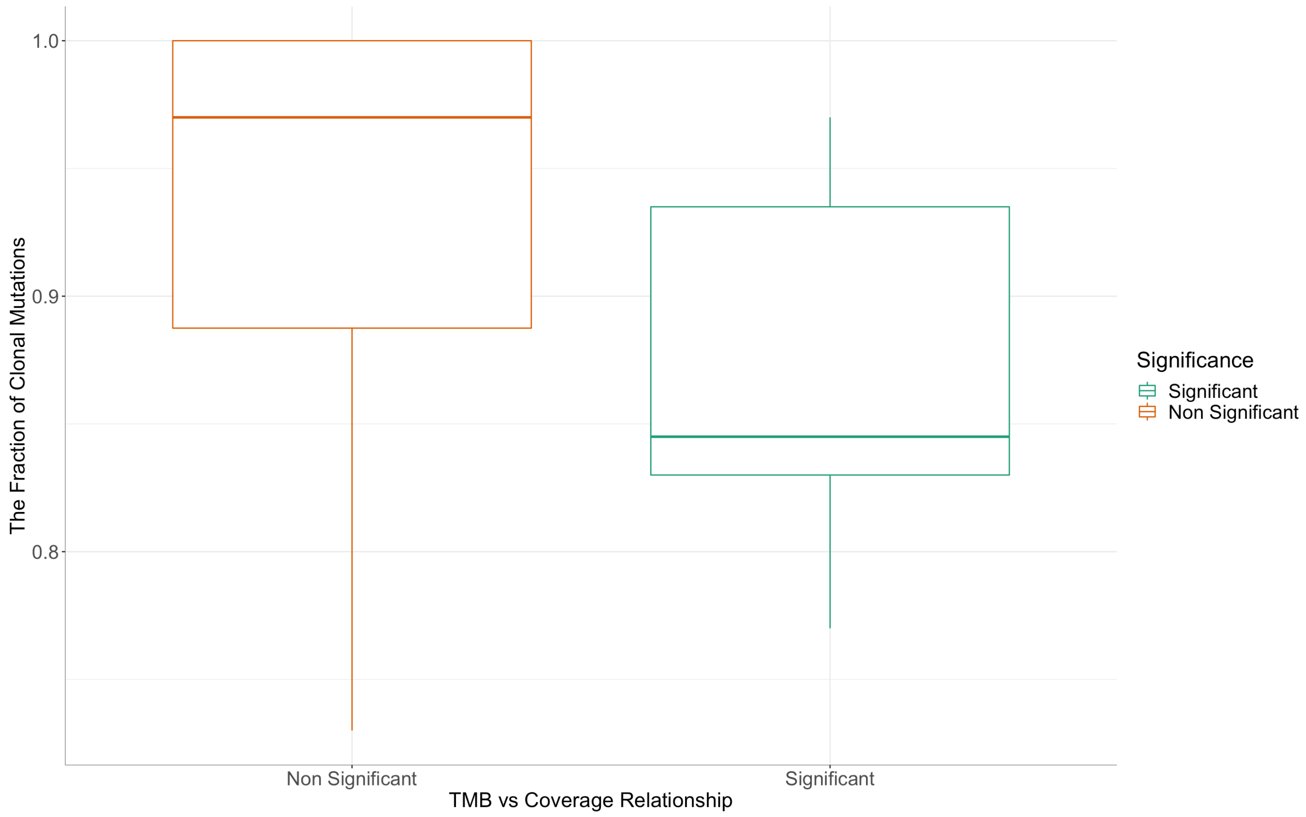

Fig. S7: In cancer types for which the correlation between TMB and coverage were found significant, the fraction of samples with high clonal proportion is generally lower. This suggest that the lack of correlation between TMB and mean coverage is due to the high clonal proportion.
